# Streamline automated biomedical discoveries with agentic bioinformatics

**DOI:** 10.1093/bib/bbaf505

**Published:** 2025-09-28

**Authors:** Juexiao Zhou, Jindong Jiang, Zhongyi Han, Zijian Wang, Xin Gao

**Affiliations:** Syneron Opal, 10281, Cayman Island; Computer Science Program, Computer, Electrical and Mathematical Sciences and Engineering Division, King Abdullah University of Science and Technology (KAUST), Thuwal 23955-6900, Kingdom of Saudi Arabia; Center of Excellence for Smart Health, King Abdullah University of Science and Technology (KAUST), Thuwal 23955-6900, Kingdom of Saudi Arabia; Center of Excellence on Generative AI, King Abdullah University of Science and Technology (KAUST), Thuwal 23955-6900, Kingdom of Saudi Arabia; School of Data Science, The Chinese University of Hong Kong, Shenzhen (CUHK-Shenzhen), Guangdong 518172, P.R. China; Department of Statistics, Nanjing University, Nanjing 210008, China; Computer Science Program, Computer, Electrical and Mathematical Sciences and Engineering Division, King Abdullah University of Science and Technology (KAUST), Thuwal 23955-6900, Kingdom of Saudi Arabia; Center of Excellence for Smart Health, King Abdullah University of Science and Technology (KAUST), Thuwal 23955-6900, Kingdom of Saudi Arabia; Center of Excellence on Generative AI, King Abdullah University of Science and Technology (KAUST), Thuwal 23955-6900, Kingdom of Saudi Arabia; School of Software, Shandong University, Jinan 250101, China; School of Data Science, The Chinese University of Hong Kong, Shenzhen (CUHK-Shenzhen), Guangdong 518172, P.R. China; Computer Science Program, Computer, Electrical and Mathematical Sciences and Engineering Division, King Abdullah University of Science and Technology (KAUST), Thuwal 23955-6900, Kingdom of Saudi Arabia; Center of Excellence for Smart Health, King Abdullah University of Science and Technology (KAUST), Thuwal 23955-6900, Kingdom of Saudi Arabia; Center of Excellence on Generative AI, King Abdullah University of Science and Technology (KAUST), Thuwal 23955-6900, Kingdom of Saudi Arabia

**Keywords:** AI agents, bioinformatics, large language models

## Abstract

The emergence of artificial intelligence agents powered by large language models marks a transformative shift in computational biology. In this new paradigm, autonomous, adaptive, and intelligent agents are deployed to tackle complex biological challenges, leading to a new research field named agentic bioinformatics. Here, we explore the core principles, evolving methodologies, and diverse applications of agentic bioinformatics. We examine how agentic bioinformatics systems work synergistically to facilitate data-driven decision-making and enable self-directed exploration of biological datasets. Furthermore, we highlight the integration of agentic frameworks in key areas such as personalized medicine, drug discovery, and synthetic biology, illustrating their potential to revolutionize healthcare and biotechnology. In addition, we address the ethical, technical, and scalability challenges associated with agentic bioinformatics, identifying key opportunities for future advancements. By emphasizing the importance of interdisciplinary collaboration and innovation, we envision agentic bioinformatics as a major force in overcoming the grand challenges of modern biology, ultimately advancing both research and clinical applications.

## Main

Bioinformatics, a discipline rooted at the intersection of biology, computer science, and mathematics, has undergone remarkable evolution since its inception in the mid-20th century [1]. Initially emerging as a computational response to the increasing complexity of biological data [2], bioinformatics was propelled by breakthroughs in molecular biology and the development of sequencing technologies [3]. Early milestones, such as the development of the sequence alignment algorithms [4] and the establishment of pioneering genetic databases [5, 6], laid the foundation for a field that would transform our understanding of life. Over the decades, bioinformatics has expanded its scope far beyond genomics, encompassing proteomics, transcriptomics, metabolomics, and systems biology [7–10]. This growth has been paralleled by significant advancements in computational power, algorithm design, and data storage, enabling the integration of machine learning and high-performance computing into bioinformatics workflows. These technological innovations have facilitated the exploration of intricate biological networks and the modeling of cellular systems, ushering in an era of predictive and system-level analyses [11, 12].

Today, bioinformatics stands as the cornerstone of modern biology and medicine, driving discoveries in areas ranging from evolutionary biology and structural genomics to personalized medicine and synthetic biology [13]. The rapid evolution of artificial intelligence (AI) and deep learning (DL) has profoundly affected numerous scientific disciplines [14], with bioinformatics being no exception [15–17]. From the early conceptualization of AI to the emergence of large language models (LLMs) [18] and LLM-driven AI agents [19], AI has transitioned from rule-based systems to advanced models capable of understanding, reasoning, and interacting with complex datasets. LLM-driven AI agents, which are autonomous systems designed to perceive environments, make decisions, and execute actions, represent a critical milestone in this evolution [20]. Their integration into bioinformatics marks a paradigm shift, characterized by the use of intelligent agents to autonomously generate, analyze, and interpret biological data. This approach addresses the growing scale and complexity of biological research, unlocking new possibilities for innovation and collaboration [21].

The use of agent-based approaches in bioinformatics has a well-established history. As early as the NETTAB 2001 and 2002 workshops [22, 23], researchers explored intelligent agents to enhance interoperability, data integration, and decision-making across distributed biological databases. Notably, Merelli *et al*. [24] introduced one of the first agent-based systems for automating workflows and processing DNA microarray data. While today’s focus has shifted toward LLM-based agents capable of natural language reasoning and high-level task planning, these modern systems still build upon the original vision of autonomous, interoperable agents in computational biology [25].

Conventional bioinformatics approaches have relied heavily on manual curation and rigid workflows [26–28]. While these methods have delivered significant insights, they are increasingly limited by their scalability, adaptability, and ability to manage the rapidly expanding volume and heterogeneity of biological datasets, as well as the demands of emerging research pipelines [29]. These challenges highlight the pressing need for more dynamic and intelligent solutions. AI agents, with their transformative potential, offer a promising path forward. They enable capabilities such as real-time data generation, autonomous experimental design, and high-dimensional data analysis, addressing the limitations of traditional methods and unlocking new possibilities in bioinformatics [21, 29, 30].

Thus, we define and envision **agentic bioinformatics** as a novel and transformative paradigm within bioinformatics, wherein intelligent AI agents are strategically integrated throughout the entire research process to optimize, automate, and innovate biological data analysis. Agentic bioinformatics goes beyond the mere application of LLMs or isolated AI agents in bioinformatics workflows. It emphasizes end-to-end integration of intelligent, autonomous agents that can reason, plan, adapt, and collaborate across the entire scientific process. These agents leverage advanced machine learning, natural language processing, and autonomous decision-making techniques to assume diverse, dynamic roles across the bioinformatics pipeline, ranging from data preprocessing and analysis to result interpretation and hypothesis generation. These agents are designed not just to execute predefined tasks, but to dynamically coordinate with one another, make independent decisions under uncertainty, and engage in long-horizon planning tailored to the complexities of biological systems. By facilitating end-to-end automation and offering innovative solutions, agentic bioinformatics enables more efficient and insightful scientific discovery in biology.

Agentic bioinformatics represents a fundamental shift that introduces a multi-agent, adaptive framework that aligns more closely with the open-ended, exploratory nature of biological research, where goals evolve and data contexts shift. Tasks that once demanded extensive human expertise and time are now streamlined, facilitating rapid hypothesis iteration and validation. Furthermore, this paradigm promotes inclusivity by democratizing access to advanced analytical tools, allowing researchers with varying levels of computational expertise to leverage state-of-the-art methods. In contrast to traditional LLM-based workflow automation, agentic bioinformatics entails a system-level rethinking of how biological knowledge is generated, transforming agents from tools into autonomous collaborators capable of hypothesis generation, experimental design, and iterative refinement. This systemic view enables enhanced scalability, inclusivity, and accessibility, allowing researchers with diverse backgrounds to leverage sophisticated methods through intelligent mediation. By bridging disciplines, agentic bioinformatics could empower collaborative efforts across the biological sciences, fostering innovation and accelerating discovery.

Here, we comprehensively explore the concept and implications of agentic bioinformatics, with key terminology defined in Table 1. We begin with an overview of existing operational frameworks of AI agents in bioinformatics, distinguishing between single-agent and multi-agent systems. Single-agent systems focus on specialized, independent tasks, while multi-agent systems involve collaboration and task distribution across agents. The discussion emphasizes how these systems can tackle complex biological problems more effectively than traditional methods. Next, we present a forward-looking vision for a fully automated, end-to-end laboratory powered by agentic bioinformatics. In such a laboratory, intelligent agents manage every stage of the research process, from hypothesis generation and experimental design to data analysis, interpretation, and reporting. This vision integrates wet-lab robotics, dry-lab computational agents, and real-time decision-making systems, revolutionizing the pace and scope of biological discovery. Finally, we address the opportunities and challenges associated with agentic bioinformatics. Opportunities include enhancing reproducibility, scalability, and innovation in research, while challenges span ethical considerations, data privacy, bias mitigation, the development of robust, interpretable AI systems, among others. Our objective is to underscore the transformative potential of AI agents in bioinformatics and their critical role in advancing biological research. Agentic bioinformatics is not merely an incremental advancement, it signifies a paradigm shift that redefines the boundaries of what is possible in the life sciences. By exploring the applications, frameworks, and future directions of agentic bioinformatics, we aim to inspire new research, foster interdisciplinary collaboration, and provide a roadmap for realizing the full potential of this emerging field.

**Table 1:** Glossary of key terms in agentic bioinformatics

Term	Definition
AI agent	An autonomous or semi-autonomous computational entity capable of perceiving its environment, processing information, and taking actions to achieve specific goals. In the context of bioinformatics, AI agents operate across both wet-lab and dry-lab settings.
LLM	A type of AI model trained on large corpora of text to perform natural language processing tasks. In this framework, LLMs are used as foundational components for agents such as Literature Review Agents and Reasoning Agents, enabling sophisticated language understanding and generation.
Agentic bioinformatics	An interdisciplinary paradigm that integrates autonomous AI agents into the bioinformatics lifecycle, from hypothesis generation and experimental execution to data analysis and interpretation, across both wet-lab and dry-lab environments.
Wet-lab AI agent	An AI-driven system, often embodied in hardware or interfaced with physical lab equipment, that performs experimental tasks such as sample preparation, PCR, microscopy, or animal testing. These agents support experimental throughput, precision, and reproducibility.
Dry-lab AI agent	A software-based AI entity that handles computational tasks, including data mining, statistical analysis, machine learning, and hypothesis generation, using digital datasets such as genomic sequences or imaging data.
Search agent	A type of dry-lab agent designed to retrieve relevant scientific information from structured and unstructured databases, assisting in the knowledge-gathering phase of research.
Literature review agent	An AI agent powered by LLMs that synthesizes insights from scientific literature to support experiment planning and contextualization of results.
Database agent	A dry-lab AI agent that manages and queries large-scale biological databases, enabling efficient access to structured biological data.
Innovative AI agent	An AI agent that can design forward-thinking AI applications, such as the design of novel algorithms and the development of novel applications.
Reasoning agent	An advanced dry-lab AI agent that applies logic, inference, and probabilistic modeling to derive insights from complex datasets and guide decision-making.

## Agentic bioinformatics

**Figure 1:**
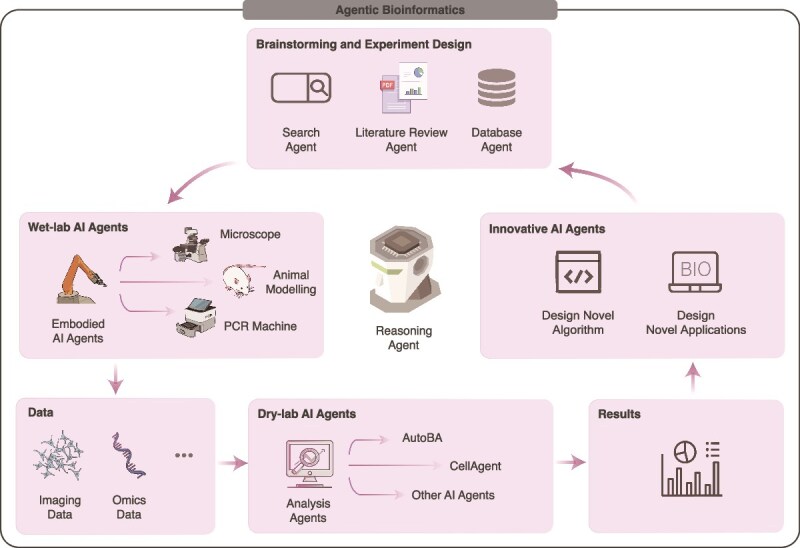
**Conceptual framework for agentic bioinformatics.** We present an interdisciplinary framework for agentic bioinformatics, which integrates AI-driven agents into both dry-lab and wet-lab environments to revolutionize bioinformatics workflows. The framework is organized into functional sections that highlight the key roles and interactions of autonomous AI agents across various stages of bioinformatics research. **Brainstorming and experiment design:** the research process begins with brainstorming and experiment design, facilitated by specialized AI agents such as Search Agents and Literature Review Agents. These agents retrieve, curate, and synthesize scientific knowledge from diverse databases, enabling researchers to generate hypotheses efficiently. By providing contextualized insights from the latest research, these tools streamline the initial stages of scientific inquiry and ensure that experiments are grounded in up-to-date evidence. **Wet-lab AI Agents:** wet-lab AI agents represent a class of physical or semi-physical AI systems that interact directly with laboratory equipment to execute experimental procedures. Examples include polymerase chain reactions (PCR) machines, microscopes, and animal modeling agents, which perform tasks such as PCR, high-resolution imaging, and *in vivo* modeling. These agents enhance precision, reduce human error, and accelerate workflows, enabling researchers to focus on higher level decision-making and interpretation. **Dry-lab AI Agents:** dry-lab AI agents, such as Database Agents and Reasoning Agents, are pivotal in managing, analyzing, and interpreting complex datasets, including genomic, imaging, and multi-omics data. Advanced AI-driven tools like AutoBA and CellAgent further refine data interpretation, extracting meaningful biological insights and uncovering patterns that may not be apparent through traditional methods. These agents bridge the gap between raw data and actionable knowledge, enabling researchers to make data-driven decisions with confidence. **Innovative AI agents:** the framework also emphasizes the role of forward-thinking AI applications, such as the Design of Novel Algorithms and the Development of Novel Applications. These innovative agents exemplify the creative potential of AI in bioinformatics, enabling the exploration of uncharted territories and the development of groundbreaking tools. By pushing the boundaries of what is possible, these agents facilitate discoveries and applications that were previously unattainable. The seamless integration of wet-lab and dry-lab agents creates a collaborative ecosystem where AI accelerates the pace of bioinformatics research. By synergizing embodied agents (wet-lab) and computational agents (dry-lab), the framework enables a continuous flow from data generation to analysis and interpretation. This holistic approach addresses complex biological questions with unprecedented sophistication, paving the way for transformative advancements in the field.

As illustrated in Fig. 1, intelligent agents in bioinformatics can function in a variety of roles, each tailored to address specific aspects of biological research and data analysis. These roles include, but are not limited to Brainstorming Agents, which assist in hypothesis generation and ideation; Experimental Design Agents, which optimize research workflows and suggest experimental parameters; Reasoning Agents, which draw inferences and establish causal relationships from data; Wet-lab AI agents, which control and automate laboratory equipment for physical experiments; Dry-lab AI agents, which focus on computational analysis and simulations; and Innovative AI agents, which explore novel strategies and generate creative solutions to complex problems. Together, these agents form a versatile and adaptive toolkit for agentic bioinformatics, enabling researchers to address diverse challenges across the biological research spectrum.

Agents in bioinformatics operate under two primary paradigms: single-agent systems and multi-agent systems. Each paradigm serves distinct purposes and offers unique advantages depending on the complexity and scope of the task. Single-agent systems comprise a stand-alone AI agent that executes specific tasks independently. They are designed for high specialization and focus, excelling in tasks that require in-depth expertise or precision. Such systems are particularly effective for compartmentalized tasks that can be solved in isolation, offering simplicity and efficiency in implementation. However, their stand-alone nature limits their ability to address interconnected or multifaceted problems.

In contrast, multi-agent systems consist of multiple intelligent agents working collaboratively to tackle complex challenges. These systems are characterized by their ability to distribute responsibilities, coordinate actions, and adapt dynamically to evolving tasks. The adaptability and scalability of multi-agent systems make them ideal for addressing the intricate and dynamic nature of modern bioinformatics challenges. They enable researchers to model complex systems, explore multidimensional datasets, and solve problems that exceed the capabilities of stand-alone agents.

By leveraging the strengths of both single-agent and multi-agent systems, bioinformatics researchers can tailor their approaches to the specific demands of their work, ensuring optimal efficiency and innovation.

### Single AI agent in bioinformatics

Single agent in bioinformatics is typically designed to handle specific tasks such as data analysis or experiment control. These agents have proven highly effective in managing well-defined tasks, offering significant advantages in simplicity, cost-effectiveness, and task specialization, as shown in Table 2. A comparative analysis of their shared and distinct characteristics is provided in Table 3.

**Table 2:** Single-agent systems for agentic bioinformatics. An asterisk (*) indicates a non-peer-reviewed paper

Method name	Year	Journal	Model	Task	Key feature	Reproducibility
BioMANIA	2023	bioRxiv*	GPT4	omics data analysis	A chatbot generation pipeline with a user-friendly back-end service for seamless interaction	https://github.com/batmen-lab/BioMANIA
AutoBA	2023	Advanced Science	Multiple LLMs	omics data analysis	Automated multi-omics analysis with minimal user input while providing detailed, step-by-step analysis plans	https://github.com/JoshuaChou2018/AutoBA
BioInformatics Agent (BIA)	2024	bioRxiv*	GPT4	scRNA-seq data analysis	Autonomous bioinformatic analysis through natural language	https://github.com/biagent-dev/bia
BRAD	2024	Bioinformatics	GPT4	Various biological tasks	Enable tasks such as Retrieval-Augmented Generation, searches across bioinformatics databases, and the execution of software pipelines	https://github.com/jpickard1/brad
LM-ABC	2024	bioRxiv*	GPT4	enzyme engineering	A computational tool that merges LLMs with biocatalysis-specific modules to streamline enzyme engineering	https://github.com/GT4SD/lm-assistant-for-biocatalysis
CRISPR-GPT	2024	bioRxiv*	GPT4	CRISPR genome engineering	Assist nonexpert researchers in designing gene-editing experiments and validates effectiveness in real-world use cases	https://github.com/cong-lab/crispr-gpt-pub
BioDiscoveryAgent	2024	ICLR	Claude 3.5 Sonnet	design genetic perturbation experiments	Design new experiments, reasons about their outcomes, and efficiently navigates the hypothesis space to reach desired solutions	https://github.com/snap-stanford/BioDiscoveryAgent
SpatialAgent	2025	bioRxiv*	GPT-4o, Claude 3.5 Sonnet	conduct spatial genomics research	Process multimodal inputs, incorporate external databases, and support human-in-the-loop interactions, enabling both fully automated and collaborative discovery	https://github.com/Genentech/SpatialAgent
MDCrow	2025	arXiv*	GPT-4o, llama3-405b	Molecular dynamics simulations	Consist of an environment of tools that emit observations and an LLM that selects actions	https://github.com/ur-whitelab/MDCrow

**Table 3:** **Feature comparison of single-agent systems for agentic bioinformatics**. Automated: the agent completes a self-contained task without human intervention. Human interaction: the agent follows user commands or instructions. Experiment design: the agent is capable of independently designing experimental protocols relevant to its task. Data analysis: the agent performs statistical computations, pattern recognition, and derives insights from raw datasets

Method name	Automated	Human interaction	Experiment design	Data analysis
BioMANIA	$\times $	$\checkmark $	$\times $	$\checkmark $
AutoBA	$\checkmark $	$\times $	$\times $	$\checkmark $
BioInformatics Agent (BIA)	$\checkmark $	$\times $	$\checkmark $	$\checkmark $
BRAD	$\checkmark $	$\times $	$\times $	$\checkmark $
LM-ABC	$\times $	$\checkmark $	$\times $	$\times $
CRISPR-GPT	$\checkmark $	$\checkmark $	$\checkmark $	$\times $
BioDiscoveryAgent	$\checkmark $	$\times $	$\checkmark $	$\checkmark $
SpatialAgent	$\checkmark $	$\checkmark $	$\checkmark $	$\checkmark $
MDCrow	$\times $	$\checkmark $	$\times $	$\checkmark $

Traditional bioinformatics data analysis requires multiple tools and substantial programming expertise, which limits accessibility to experimental researchers due to the steep learning curve. BioMANIA [31] combines LLMs with Application Programming Interfaces (APIs) from established Python libraries to streamline high-throughput sequencing data analysis, addressing the complexities and technical challenges inherent in these tasks. By interpreting user instructions and automatically executing bioinformatics workflows, BioMANIA enables code-free biological analyses, simplifying omics data exploration and accelerating research. Despite these advantages, BioMANIA still faces limitations with ambiguous instructions and heavily relies on high-quality documentation. Its performance is limited by reliance on the quality of third-party tools, encountering challenges such as installation failures, undocumented dependencies, and inconsistencies between API documentation and code. The system also struggles with poorly designed APIs, characterized by ambiguous names and excessive parameters, and unreliable tutorials, while remaining susceptible to LLM hallucinations during API prediction.

BIA [32] provides an interactive, automated solution for single-cell RNA sequencing (scRNA-seq) analysis, leveraging text-based interactions with LLMs to facilitate data extraction, analysis, and report generation through dynamic user dialog. The agent executes the entire single-cell analysis pipeline, from data retrieval to invoking the necessary APIs for processing, and autonomously plans and compiles conclusions. However, the system’s performance in zero-shot dynamic workflows reveals notable limitations, including frequent generation of incomplete experimental designs that omit critical steps like subcluster annotation because of the lack of professional knowledge, inconsistent tool recommendations for identical queries indicating underlying stability issues, and a persistent need for manual intervention due to inadequate autonomous refinement capabilities.

AutoBA [18] autonomously handles multi-omics data analysis with remarkable ease of use. By requiring minimal user input, such as the data path, description, and analysis goal, AutoBA autonomously proposes an analysis plan, generates and executes the necessary code, and performs the subsequent data analysis. For example, in a task focused on identifying differentially expressed genes, the user provides RNA-Seq data, along with its description and analysis goal. AutoBA then automatically performs the required preprocessing steps and generates an analysis table as output. Its ability to adapt and self-design analysis processes based on variations in input data and the novel automated code repairing module further enhances its flexibility and versatility. While demonstrating robust performance across 40 test cases, the system’s generalizability requires further validation given the vast diversity of tasks in classical bioinformatics analysis.

BRAD [33] is used for automation, performing tasks ranging from gene enrichment and archival searches to automatically generating code for biomarker identification pipelines. It is organized into several specialized modules: the LAB NOTEBOOK module for literature searches, the SOFTWARE module for software generation and execution, and the DIGITAL LIBRARY module for database and web searches. Bioinformatics tools like LM-ABC [34] not only accelerate enzyme engineering workflows through dynamic selection of specialized tools for tasks such as binding site extraction, catalytic activity optimization, and molecular dynamics simulations but also face implementation challenges when generated code snippets or workflows encounter failures stemming from incomplete or erroneous tool integration. By combining AI-guided molecular design with robust interoperability validation, these systems aim to balance automated experimental optimization with technical reliability, enabling researchers to navigate both biological complexity and computational constraints in enzyme engineering pipelines. But its effectiveness remains unquantified due to the absence of benchmark comparisons that would establish its success rate, accuracy, and other key performance indicators.

CRISPRGPT [35] streamlines gene-editing research by automating the design of CRISPR systems, guide RNAs, delivery methods, and validation protocols, while its reliance on curated biological databases introduces limitations in scenarios where species-specific genomic data or gene annotations are incomplete. The system’s ability to democratize experimental design for nonexperts is inherently tied to the coverage and accuracy of its underlying knowledge resources, necessitating iterative updates to address gaps in underrepresented organisms or emerging gene targets. This integration of automation with domain-specific constraints highlights both the transformative potential and context-dependent challenges of AI-augmented workflows in genome engineering. Safety and ethical concerns may arise when using AI tools to guide genome editing, including risks such as unauthorized modification of human genomes and privacy breaches involving users’ genomic data.

In spatial genomics research, autonomous agents hold significant potential to drive innovation. SpatialAgent [36] exemplifies this promise by autonomously executing various spatial biology tasks, from experimental design and multimodal data analysis to hypothesis generation. Its adaptive reasoning engine and dynamic tool integration enable it to work effectively across diverse datasets, tissue types, and biological questions. Notably, it preforms strongly in large-scale, spatially resolved cell and tissue niche annotation. However, evaluation results suggest a tendency to rely on common annotation patterns from its training data rather than fully adapting to novel biological contexts. This contrasts with domain experts, who often incorporate subtle, context-specific cues during manual annotation.

However, while the simplicity and specialization of single agents offer clear benefits, they also present distinct challenges. Focusing on specific tasks can limit their ability to handle complex, multifaceted problems that require cross-domain knowledge or collaboration between multiple agents. Moreover, the lack of synergy between isolated agents can lead to inefficiencies, particularly when dealing with tasks that require multiple steps or diverse methodologies.

### Multi AI agents in bioinformatics

Multi-agent systems have emerged as indispensable tools for tackling complex biological problems. These challenges include data processing in genomics, drug discovery, and protein design. By collaborating, different agents can effectively share tasks, improve productivity, and adapt to new challenges. This section will explore the collaboration models of multi-agent systems in bioinformatics, focusing on collaborative task-solving, dynamic task allocation, and continuous learning and adaptability (Table 4). The comparative analysis of their shared and distinct characteristics is provided in Table 5.

**Table 4:** Multi-agent systems for agentic bioinformatics. An asterisk (*) indicates a non-peer-reviewed paper

Method name	Year	Journal	Model	Task	Key feature	Reproducibility
CellAgent	2024	bioRxiv*	GPT4, GPT-4V	scRNA-seq data analysis	A hierarchical decision-making mechanism to coordinate these biological experts, effectively driving the planning and step-by-step execution of complex data analysis tasks	http://cell.agent4science.cn
The Virtual Lab	2024	bioRxiv*	GPT4	Design nanobody binders to recent variants of SARS-CoV-2	An LLM principal investigator agent guiding a team of LLM agents with different scientific backgrounds, with a human researcher providing high-level feedback	https://github.com/zou-group/virtual-lab
Bio-Copilot	2024	bioRxiv*	Multiple LLMs	Large-scale omics studies	Bio-Copilot decomposes a bioinformatics task into modular, hierarchical steps, configures agent groups, and specifies roles according to step characteristics, while formulating rules for task allocation and scheduling. Researchers collaborate with agent groups to generate explicit execution plans, and execution agent groups gather resources and carry out plans	https://github.com/lyyang01/Bio-Copilot
ProtAgents	2024	Digital Discovery	GPT-4	Protein design and analysis	A platform for de novo protein design based on LLMs, where multiple AI agents with distinct capabilities collaboratively address complex tasks within a dynamic environment	https://github.com/lamm-mit/ProtAgents
BioMaster	2025	bioRXiv*	GPT-4	Various bioinformatics tasks	Integration of a tailored memory mechanism and specialized agents to optimize input/output handling and management in complex bioinformatics workflows	https://github.com/ai4nucleome/BioMaster
BioAgents	2025	arXiv*	Phi-3	Assist users in designing complex bioinformatics pipelines	Multi-agent system built on small language models, fine-tuned on bioinformatics data, and enhanced with retrieval augmented generation	close-sourced

**Table 5:** **Feature comparison of multi-agent systems for agentic bioinformatics**. Specialized roles: agents are assigned distinct, complementary roles within the system. Self-optimization: the system utilizes performance feedback to enable self-tuning and adaptive optimization. Human-guided collaboration: the system incorporates scientific expertise through structured mechanisms for human–agent interaction

Method name	Specialized roles	Self-optimization	Human-guided collaboration
CellAgent	$\checkmark $	$\checkmark $	$\times $
The Virtual Lab	$\checkmark $	$\checkmark $	$\checkmark $
Bio-Copilot	$\checkmark $	$\checkmark $	$\checkmark $
ProtAgents	$\checkmark $	$\checkmark $	$\times $
BioMaster	$\checkmark $	$\checkmark $	$\times $
BioAgents	$\times $	$\checkmark $	$\checkmark $

Multiple agents work together to solve complex tasks, often by decomposing them into smaller sub-tasks and distributing them among different agents. In the CellAgent [37] framework, three agents, the Planner, Executor, and Evaluator, each take on different roles to collaboratively analyze scRNA-seq data. The Planner designs a comprehensive analysis workflow, the Executor executes the designated tasks, and the Evaluator ensures accuracy and biological relevance by conducting rigorous assessments. BioMaster [38] employs a similar multi-agent design, where specialized agents collaborate to plan, code, and debug, enabling seamless execution of complex tasks such as Hi-C data processing.

In complex bioinformatics tasks, new challenges and unforeseen issues may arise during the experiment. Multi-agent systems are equipped with continuous learning and adaptability mechanisms to optimize their strategies and behaviors over time. CellAgent also incorporates a self-iterative optimization mechanism that integrates automated evaluation results and adjusts the execution strategy to address potential technical problems or experimental anomalies. This self-improvement capability enables the system to adapt to changes and maintain high efficiency in execution quickly. However, CellAgent also faces several challenges. Its memory systems can become excessively large, leading to performance degradation in LLMs when handling overly long contexts. CellAgent’s main limitation lies in its restricted self-evaluation capability. While the GPT-4-based evaluation enables autonomous optimization, it currently lacks flexibility for diverse analytical objectives, requiring manual adjustment for specialized needs.

Multi-agent systems often need to dynamically allocate tasks based on the complexity, priority, and real-time needs of the tasks, enhancing system efficiency and ensuring optimal resource utilization. In the Bio-Copilot framework [39], the task distribution is based on the expertise of each agent and the current demands of the system, with a coordination layer ensuring smooth execution and real-time feedback allowing adjustments to task allocation for improved collaborative efficiency. Bio-Copilot’s multi-agent architecture presents two key limitations. First, its competitive learning mechanism, while effective for performance optimization through parallel task execution and cross-evaluation, incurs significant computational costs in resource allocation and energy consumption. Second, the system’s prompt engineering faces challenges in multi-agent coordination, where ambiguous or context-poor instructions can compromise decision-making accuracy.

Multi-agent systems have been successfully applied to a wide range of bioinformatics tasks. For example, ProtAgent [40] employs a multi-agent framework to streamline protein design and analysis through role specialization while automating the selection and deployment of external bioinformatics tools. Each agent has a specific function: the Planner formulates strategies, the Executor automatically identifies and applies appropriate tools from external libraries for specific bioinformatics tasks, and the Critic continuously assesses the effectiveness and appropriateness of the tools being used. This system is engineered to dynamically select and evaluate the most suitable tools based on task requirements, ensuring that ProtAgent not only adapts seamlessly across various protein design scenarios but also enhances other functionalities integral to the protein engineering process. ProAgent demonstrates constrained capabilities in multi-objective protein design, as its current architecture primarily employs data-driven end-to-end DL models. While effective for individual characteristic prediction, the system struggles to optimize competing design requirements simultaneously. This limitation becomes particularly evident when addressing complex biological specifications that demand concurrently balancing multiple structural and functional parameters.

The Virtual Lab [41] functions as a collaborative platform where AI and human expertise converge. Guided by a principal investigator AI agent, the system coordinates a diverse team of AI agents with expertise in fields like chemistry and computer science, complemented by a human researcher who provides high-level feedback. This collaborative setup is structured through a series of team and individual meetings. Team meetings facilitate collaborative discussions on overarching scientific goals, whereas individual meetings focus on delegating and clarifying agent-specific tasks. The Virtual Lab effectively addresses complex, real-world scientific challenges, such as the development of nanobody binders targeting new SARS-CoV-2 variants. In the experiments, it successfully validated 92 engineered nanobodies, with over 90% exhibiting expression and solubility. In particular, two of these nanobodies showed unique binding properties to recent JN.1 and KP.3 spike RBD variants, highlighting the Virtual Lab’s innovative capacity to drive scientific breakthroughs. But the Virtual Lab requires repeated refinements to achieve optimal results, increasing time costs and facing challenges with efficient resource utilization and ambiguous prompts. The Virtual Lab’s effectiveness is constrained by inherent LLM limitations, particularly in temporal knowledge currency and decision-making precision. Its AI agents occasionally recommend outdated tools (like AlphaFold-Multimer instead of AlphaFold3) due to training data cutoffs, and require human intervention to correct deprecated code implementations. Furthermore, the system exhibits prompt sensitivity, when faced with binary design choices (e.g. nanobody modification vs. de novo design), agents often default to noncommittal responses unless explicitly constrained. These constraints currently necessitate significant human work to maintain scientific rigor.

## Challenges of agentic bioinformatics

Despite its transformative potential, agentic bioinformatics faces several critical challenges that must be addressed to ensure its effective deployment in biological research. These challenges span technical, ethical, and collaborative aspects, each of which requires targeted solutions. Below, we outline the key challenges specific to agentic bioinformatics.

### Integration and standardization

Agentic bioinformatics systems rely on a diverse ecosystem of AI agents, bioinformatics tools, and laboratory automation devices. However, the lack of standardized protocols for communication between AI-driven components impedes seamless interoperability. Existing bioinformatics workflows use heterogeneous data formats, software environments, and computational frameworks, making it difficult to integrate AI agents across different platforms. Standardization of APIs, data exchange formats, and agent communication protocols is essential to enable collaborative AI agents that operate across multiple bioinformatics tasks [42].

### High-dimensional biological data and multimodal integration

Biological data are inherently high-dimensional, spanning genomics, transcriptomics, proteomics, metabolomics, and imaging modalities [43]. Agentic bioinformatics systems must efficiently process and integrate multi-modal datasets, each with different statistical distributions and noise characteristics. Traditional bioinformatics pipelines often rely on domain-specific preprocessing steps that may not generalize well to AI-driven workflows. AI agents must be equipped with adaptive data preprocessing, feature selection, and dimensionality reduction techniques to extract meaningful patterns from complex biological datasets.

### Out-of-distribution generalization and anomaly detection

A significant challenge for AI-driven bioinformatics is generalization to unseen data distributions, particularly when analyzing datasets from different species, cell types, or experimental conditions [44]. AI agents trained on specific datasets may fail to generalize when applied to new biological contexts, leading to unreliable predictions. Out-of-distribution generalization and anomaly detection methods must be incorporated into agentic bioinformatics workflows to ensure robustness [45, 46]. This is particularly critical in clinical applications where AI-driven diagnostics and personalized medicine decisions must be validated across diverse patient populations [47].

### Hallucinations in AI-driven bioinformatics

AI agents, particularly LLMs and generative AI systems, may produce biologically implausible results or hallucinations when making predictions or generating hypotheses. In bioinformatics, hallucinations could manifest as incorrect sequence alignments, erroneous functional annotations, or false protein–ligand interactions. Unlike hallucinations in natural language processing, which are often a minor inconvenience, incorrect AI-driven bioinformatics predictions can mislead experimental designs and result in wasted resources. Implementing confidence scoring mechanisms, uncertainty quantification, and expert validation is crucial to mitigating AI hallucinations in bioinformatics [18]. Recent studies have demonstrated that medical LLMs can produce clinically unsupported content with high linguistic fluency [48, 49]. In domains like protein design, such hallucinations, e.g. inaccurate structural or binding predictions, could lead to costly downstream failures in wet-lab experiments. To mitigate these risks, methods such as retrieval-augmented generation [50] and multi-agent cross-validation [51] have been proposed, introducing external factual grounding and structured reasoning workflows to enhance reliability.

### Bias, inclusivity, and security

Bioinformatics datasets are often biased due to limited sample diversity in publicly available repositories. AI agents trained on biased datasets may produce unfair or misleading conclusions, particularly in applications such as disease biomarker discovery and drug response predictions. Bias mitigation strategies, such as adversarial debiasing and fairness-aware learning, are necessary to ensure that AI-driven insights are equitable across diverse populations [52]. In particular, ancestry-related biases in genomic databases have been widely reported to reduce predictive performance for underrepresented populations [53]. In multi-agent settings, fairness-aware coordination strategies [54] can help ensure equitable outcomes across subgroup-specific agents. Additionally, AI-driven bioinformatics workflows must address security concerns, including adversarial attacks on biological datasets and unauthorized data access. This is especially important in federated or cloud-deployed agentic systems, where data poisoning or backdoor attacks could compromise downstream scientific outcomes [55].

### Data privacy and limited annotations

Many bioinformatics applications involve sensitive genomic and clinical data, raising concerns about data privacy and regulatory compliance. Ensuring that AI agents comply with data protection regulations (e.g. GDPR, HIPAA) is crucial for their adoption in biomedical research. Federated learning and privacy-preserving AI techniques, such as differential privacy and homomorphic encryption, can help mitigate data privacy risks [56]. Recent advancements demonstrate secure multiparty computation frameworks for genome-wide association studies that preserve individual privacy without sacrificing utility [57]. Additionally, limited annotations in biological datasets pose challenges for supervised learning models, necessitating the development of weakly supervised, self-supervised, and semi-supervised learning approaches. Agentic frameworks can benefit from self-supervised pretraining on large unlabeled datasets followed by few-shot fine-tuning for specialized downstream tasks [58].

### Interpretability and explainability in AI-driven bioinformatics

Interpretability remains a major bottleneck in deploying AI-driven bioinformatics models in real-world applications. Many DL-based bioinformatics models function as black boxes, making it difficult for researchers to understand the reasoning behind their predictions. AI agents used in bioinformatics must incorporate explainability techniques to improve model transparency [59]. For instance, model attribution tools such as SHAP and Integrated Gradients are increasingly used to explain omics-based classifiers [60, 61]. In multi-agent settings, it is equally important to provide step-wise provenance logs that allow users to trace decisions across the agent graph. Explainable AI (XAI) is particularly crucial for applications like drug discovery, where the rationale behind molecule–target interactions must be validated before proceeding to experimental validation.

### Ethical considerations

The integration of AI agents in bioinformatics raises several ethical concerns. AI-generated biological hypotheses and experimental designs must be evaluated to ensure that they do not lead to unintended consequences, such as unethical genetic modifications or dual-use research applications. Ethical AI frameworks should be developed to guide the responsible use of AI agents in bioinformatics research, ensuring alignment with bioethical principles [62]. For example, recent work has demonstrated how generative models can be repurposed to design toxic molecules in silico [63], raising the urgency of adding explicit safeguards. Integrating kill switches, access constraints, and auditability are necessary for safe agent deployment [21].

### Human–AI collaboration in bioinformatics

AI-driven bioinformatics should augment, rather than replace human expertise. However, achieving effective human–AI collaboration remains a challenge due to the steep learning curve associated with AI-driven tools. Researchers must be trained to interact with AI agents, interpret AI-driven insights, and validate computational predictions experimentally. Additionally, AI systems must be designed to communicate their findings in a manner that is comprehensible to domain experts with varying levels of computational expertise [64]. Interactive agents that support natural language explanation, backtracking, and what-if analysis have shown promise in reducing cognitive barriers and improving user trust [65]. In particular, explainable dialog agents that allow clinicians or biologists to question AI decisions are gaining attention in biomedical domains [21].

## Methods

### Selection of agentic systems

As the notion of agentic systems in bioinformatics is still nascent but rapidly advancing, the body of literature explicitly using this terminology remains limited. Therefore, we adopted an exploratory and integrative survey methodology aimed at capturing the current landscape of relevant technologies.

We performed a targeted search of relevant literature from January 2020 to March 2025 using major academic and preprint databases, including PubMed, arXiv, bioRxiv, Google Scholar, and IEEE Xplore. Our search employed combinations of the following keywords: “AI agent,” “autonomous system,” “LLM in biology,” “wet-lab automation,” “robot scientist,” “bioinformatics pipeline,” “multi-agent system,” and “agent-based AI in science”.

We included studies, tools, and platforms that met the following criteria:

1. Explicit or implicit use of autonomous or semi-autonomous AI systems that support tasks across the bioinformatics workflow.
2. Relevance to biological data acquisition, analysis, or interpretation in either wet-lab or dry-lab contexts.
3. Availability through peer-reviewed publications, preprints, or reputable open-source platforms with community recognition.
4. Systems that exhibit agentic characteristics, such as goal-directed behavior, contextual reasoning, perception-action loops, or integration with physical or digital environments.

## Discussion

The prospect of an end-to-end automated biological discovery system powered by agentic bioinformatics represents a revolutionary shift in how biological research is conducted. In this forward-thinking model, intelligent AI agents are fully integrated throughout the research pipeline, autonomously managing every phase, from data generation and experimental design to execution, data acquisition, and analysis. This transformation has the potential to significantly reduce the time and resources needed for scientific discovery while increasing precision, reproducibility, and scalability. However, realizing this vision requires addressing the diversity of biological research, the iterative nature of experimental design, and the technical and ethical challenges of integrating AI into laboratory workflows.

### The AI-driven laboratory: a vision for automation

In the envisioned AI-driven laboratory, intelligent agents operate across all stages of the experimental workflow[66, 67]. They can autonomously generate synthetic biological data to simulate complex systems, which is particularly valuable when real-world data are scarce or difficult to obtain. AI agents can also design experiments by selecting optimal methodologies, determining experimental parameters, and refining protocols based on prior results [68, 69]. During the execution phase, AI-controlled robotic systems automate tasks such as liquid handling, sample preparation, and real-time imaging [70]. This level of automation ensures precision and reduces human error. It also enables high-throughput experimentation crucial for large-scale studies. Once experiments are conducted, AI agents immediately process and analyze the resulting data, using machine learning algorithms to identify patterns, uncover novel insights, and suggest new avenues for exploration [29]. This seamless integration reduces the time between data collection and actionable results, enabling faster hypothesis testing and refinement.

### Types of experiments amenable to automation

While the potential of AI-driven laboratories is immense, it is important to recognize that biological research is highly diverse, and not all experiments are equally suited to automation. High-throughput approaches, such as multi-omics profiling, are particularly well-suited for automation due to their parallelizable nature and reliance on large-scale data generation [71]. These experiments benefit significantly from AI agents, which can efficiently manage repetitive tasks, process vast datasets, and identify patterns that human researchers might miss [29, 72]. In contrast, low-throughput experiments often require customized skills and designs, making them less amenable to full automation. For example, experiments involving complex organismal behavior or rare biological phenomena may still rely heavily on human expertise and manual intervention.

Moreover, the iterative nature of biological research poses a unique challenge for automation. Experimental design often depends on the results of prior experiments, requiring a feedback loop where hypotheses are refined and protocols are adjusted based on new data. AI agents can play a critical role in this process by rapidly analyzing results, suggesting modifications, and optimizing experimental parameters in real-time. However, this requires robust integration between AI systems and laboratory equipment, as well as the ability to adapt to unexpected outcomes [73].

### Multi-agent systems: collaboration and scalability

A key development in this paradigm is the use of multi-agent systems, where multiple AI agents collaborate to accomplish complex tasks. In such systems, each agent focuses on a specific aspect of the research process, such as data collection, analysis, or resource management. For instance, one agent may preprocess data from biological samples, while another runs machine learning algorithms to identify key biomarkers, and yet another optimizes experimental protocols in real-time. This collaborative approach allows for the efficient execution of multifaceted research tasks that would be impossible for a single agent to accomplish in isolation.

Multi-agent systems also enhance the adaptability of the research process. As new challenges or research questions arise, agents can autonomously adjust their behavior or cooperate to address emerging issues without significant human intervention. This adaptability is particularly valuable in dynamic research environments, where experimental conditions and objectives may evolve over time.

### Technical challenges and solutions

Despite the promise of AI-driven laboratories, several technical challenges must be addressed to realize their full potential. One major challenge is the integration and standardization of diverse AI systems [74]. Effective collaboration between agents requires a unified platform that can accommodate different types of AI, such as machine learning, reinforcement learning, and natural language processing. Standardized protocols for data exchange and communication between agents are essential to ensure seamless interoperability [75].

Another challenge is the integration of AI agents with physical laboratory equipment. Ensuring that AI systems can control and interact with devices such as robotic arms, sensors, and microscopes requires robust hardware-software interfaces and real-time feedback mechanisms. Advances in the Internet of Things (IoT) and edge computing may provide solutions to these challenges, enabling seamless communication between AI agents and laboratory devices.

### Ethical and regulatory considerations

The adoption of AI-driven laboratories also raises important ethical and regulatory questions. Issues such as data privacy, the interpretability of experimental results, and the transparency of AI decision-making must be carefully addressed. For example, AI-generated insights must be explainable and reproducible to maintain scientific rigor and public trust. Additionally, the use of AI in sensitive areas such as personalized medicine and genetic engineering requires robust ethical guidelines to prevent misuse and ensure equitable access to technological advancements.

### Implications for scientific discovery

The implications of fully automated, multi-agent research environments are profound. By automating routine and labor-intensive tasks, researchers can focus on higher level scientific thinking and hypothesis generation. The speed and efficiency of AI-driven experiments would enable the exploration of previously infeasible research questions, accelerating the pace of scientific discovery. Moreover, the scalability of automated systems means that the volume and complexity of experiments that can be conducted would increase exponentially, potentially revolutionizing fields such as genomics, drug discovery, and personalized medicine.

This new paradigm also has the potential to democratize access to cutting-edge experimental capabilities. Researchers with limited expertise in computational methods could harness the power of AI for their work, broadening participation in scientific research and accelerating progress across the biological sciences. By combining the strengths of AI-driven automation with human creativity and insight, the future of biological discovery holds unprecedented promise.

## Conclusion

Agentic bioinformatics has emerged as a groundbreaking paradigm that integrates AI agents into the bioinformatics pipeline, ushering in a new era of precision, efficiency, and innovation in biological research. AI agents are now playing a key role in various aspects of bioinformatics, from data generation and experimental design to data analysis and interpretation. Their ability to autonomously perform complex tasks has the potential to drastically reduce human error, accelerate discovery, and enable researchers to address questions that were previously out of reach. This development marks a significant shift in how biological research is conducted, with AI agents now complementing, and in some cases replacing, traditional human-centered workflows.

Despite the remarkable advancements, there remain significant challenges to overcome in the field of agentic bioinformatics. One of the primary hurdles is the continued improvement of AI agents’ intelligence and capabilities. Current systems often face limitations, such as biases in algorithms, poor-quality data, and challenges in generalizing across diverse biological contexts. Enhancing the robustness, adaptability, and accuracy of these AI systems will be critical in ensuring their reliability and scalability for a wide range of biological research applications. Additionally, as AI agents become more integral to bioinformatics, there is an increasing need for more advanced integration techniques that allow these systems to work seamlessly across various domains, from machine learning and data analysis to laboratory automation.

The future of agentic bioinformatics also lies in fostering cross-disciplinary collaboration. The convergence of AI, computational biology, and experimental sciences is essential for advancing the field. Realizing the full potential of AI-driven research requires close collaboration among bioinformaticians, data scientists, experimental biologists, and AI experts. This collaboration must ensure AI systems incorporate biological understanding while experimental workflows optimize AI agents’ utilization. This collaborative effort will be pivotal in overcoming the current technological limitations and will lead to the development of more intuitive, flexible, and powerful systems.

Looking ahead, we can anticipate that AI agents will become increasingly ubiquitous in bioinformatics over the next few years. As the technology continues to evolve, the role of AI agents in the field will expand from supporting individual tasks to orchestrating entire research workflows, leading to a future where end-to-end automated biological discovery is a reality. This evolution will likely revolutionize not only research but also clinical applications, particularly in areas like personalized medicine, drug discovery, and disease modeling. By automating complex processes and offering more precise, data-driven insights, AI agents will empower researchers to make faster and more informed decisions, ultimately leading to breakthroughs that have profound implications for human health and our understanding of biology.

In conclusion, agentic bioinformatics holds great promise, but achieving its full potential will require overcoming technical, ethical, and collaborative challenges. As AI technologies advance and integrate further into biological research, the field will undoubtedly experience a transformation. AI agents will play a critical role in the next wave of scientific discovery and clinical innovation.

**Key Points**

- **Agentic bioinformatics** is an emerging paradigm that leverages AI agents powered by large language models (LLMs) to autonomously analyze, interpret, and explore complex biological data.
- **Agentic bioinformatics** represents a shift from traditional, static bioinformatics workflows toward dynamic, adaptive, and scalable systems capable of self-directed biological discovery.
- **Agentic bioinformatics** has promising applications in **personalized medicine, drug discovery, and synthetic biology**, where autonomous decision-making can accelerate innovation.
- This review outlines key technical, ethical, and scalability challenges, emphasizing the need for robust infrastructure and responsible deployment of agentic systems.

## Data Availability

No new data were generated or analysed in support of this research.
